# Spiritual Well-Being, Disease Perception, and Disease Adaptation in Diabetic Patients in Southern Turkey: A Cross-Sectional Study

**DOI:** 10.7759/cureus.62659

**Published:** 2024-06-18

**Authors:** Derya Atik, Esra Keşer, Ulviye Ozcan Yuce, Songül Güngör

**Affiliations:** 1 Nursing, Osmaniye Korkut Ata University, Osmaniye, TUR

**Keywords:** diabet, adaptation, illness perception, well-being, spirituality

## Abstract

Introduction

This cross-sectional descriptive study investigated the relationship between spiritual well-being, disease perception, and disease adaptation in individuals with diabetes mellitus (DM).

Methods

The sample consisted of 340 patients admitted to the internal medicine outpatient clinics of a city hospital in southern Turkey between January 2022 and January 2023. Data were collected using patient information, the Spiritual Well-Being Scale (SWBS), the Illness Perception Questionnaire (IPQ), and the Adaptation to Chronic Illness Scale (ACIS). The data were analyzed at a significance level of 0.05.

Results

Most participants were familiar with integrative interventions (84.1%). Less than half of the participants learned about integrative interventions from friends (46%). Less than a quarter of the participants had turned to integrative interventions (23.5%), such as cupping therapy (7.6%) and cinnamon therapy (7.1%). Participants had mean SWBS and ACIS scores of 118.40±11.46 and 84.46±9.18, respectively. There was a positive correlation between the ACIS and SWBS scores. There was also a positive correlation between total SWBS scores and scores on the IPQ “perceptions about the illness” subscale “timeline (acute/chronic)”. Additionally, there was a positive correlation between the total ACIS score and the scores on the IPQ subscales “perceptions about the illness”, “personal control”, “treatment control”, and “illness identification”.

Conclusion

DM patients with greater spiritual well-being tend to adhere more to their treatment and management regimens. Moreover, DM patients with more positive perceptions of their illness tend to have greater levels of spiritual well-being, which correlates with improved adaptation to their disease management and treatment protocols.

## Introduction

Diabetes mellitus (DM) is a chronic disease that impacts various physiological systems, leading to a range of physical, social, and psychological challenges for individuals affected by the condition. The global prevalence of diabetes was recorded at 8.8% in 2015 and is projected to increase to 10.4% by 2040. Among European countries, Türkiye has the highest diabetes prevalence rate at 14.6%. The increasing population, extended life expectancy, adoption of unbalanced diets, rising rates of obesity, and reduced physical activity contribute to the annual escalation in the prevalence of diabetes [1].

Adapting to a chronic disease involves demonstrating effective coping skills, avoiding post-illness mental disorders, experiencing fewer negative emotions, and successfully maintaining pace with the required lifestyle adjustments. When patients with chronic diseases successfully adapt to their new lifestyle and adhere to medical treatments, they can achieve better control over their conditions. Conversely, failure to do so can lead to difficulties in effectively managing their diseases [2]. Adherence to treatment is influenced by a variety of factors, including socioeconomic conditions, characteristics of the health system, attributes of the disease itself, features of the treatment regimen, and individual patient-related factors [3].

Adapting to an illness is influenced by a range of factors, including physical, emotional, social, and spiritual elements [4]. Spirituality is a deeply personal and often transcendent aspect of human experience that relates to one's connection to something greater than oneself. It encompasses beliefs, values, practices, and experiences that provide individuals with a sense of purpose, meaning, and connection to the world around them. Spirituality often becomes more pronounced and relevant in times of crisis, such as when individuals are confronted with illness, stress, fear of mortality, existential questions, and a sense of hopelessness. During such challenging moments, individuals may turn to their spiritual beliefs and practices as a source of solace, meaning, and strength [5,6]. Spirituality can significantly impact how individuals perceive their diseases and approach healing. Spiritual beliefs and practices can shape a person's perspective on their illness, influencing how they interpret its meaning, their role in the experience, and their potential for recovery [4].

How individuals perceive their illness can significantly impact various aspects of their well-being and the progression of the disease [7]. Patients' and family members' perceptions about a disease can be crucial in various aspects of their experience and response. These perceptions can influence decisions about treatment, emotional reactions, and the overall process of adapting to the disease [8,9]. Patients with positive perceptions of their disease often possess better coping skills and strategies to manage their conditions effectively. These positive perceptions can empower them to approach their illnesses constructively, improving overall spiritual well-being [10-14].

Research on the intersection of spirituality, illness perception, and disease adaptation, which are critical factors influencing effective disease management, treatment, care, and education, is relatively scarce. For patients with DM to sustain their quality of life over the long term, it is essential that they maintain positive perceptions, effective adaptation, and heightened levels of spirituality. This study investigated the relationship between spiritual well-being, disease perception, and disease adherence in individuals with DM.

## Materials and methods

This study adopted a descriptive and cross-sectional research design. The data were collected in the internal medicine outpatient clinics of Adana City Hospital between January 2022 and January 2023. Data were collected from 340 patients who agreed to participate in the study within the specified dates. Power analysis was performed to determine the adequacy of the sample size. The results showed that the sample size was large enough to detect significant differences (a power of 98%). The inclusion criteria were as follows: (1) being older than 18 years, (2) having no communication problems, (3) having been diagnosed with DM at least six months prior, and (4) having no diagnosed mental disorder.

The data were collected using a patient information form, the Spiritual Well-Being Scale (SWBS), the Illness Perception Questionnaire (IPQ), and the Adaptation to Chronic Illness Scale (ACIS). The data were collected by the researcher, who conducted face-to-face interviews with all participants. Written and verbal consent was obtained from all participants.

Patient information form

The patient information form was developed by the researcher [2,4,8]. The form consisted of items on sociodemographics (age, sex, marital status, education, occupation, income, living arrangement, etc.) and health- and disease-related characteristics (tobacco and alcohol use, diagnosis time, body weight and height, comorbidities, etc.).

Spiritual Well-Being Scale

The SWBS was developed by Ekşi and Kardaş to identify how adults understand and live their lives in terms of personal, social, environmental, and transcendental aspects in line with their values and ultimate meanings. The instrument consists of 29 items rated on a five-point Likert-type scale (1 = not applicable to me at all, 2 = not applicable to me, 3 = somewhat applicable to me, 4 = quite applicable to me, 5 = completely applicable to me). The instrument has three subscales: transcendence (items 1, 4, 5, 8, 9, 12, 13, 16, 17, 20, 21, 24, 25, 27, and 29); harmony with nature (items 2, 6, 10, 14, 18, 22, and 28); and anomie (items 3, 7, 11, 15, 19, 23, and 26). The total score ranges from 29 to 145, with higher scores indicating higher levels of spiritual well-being [15]. The instrument has a Cronbach’s alpha of 0.89, which was 0.83 in the present study.

Illness Perception Questionnaire

The IPQ was developed by Weinman et al. [16] and revised by Moss-Morris et al. [17]. The instrument consists of three subscales: (1) illness identity, (2) perceptions about the illness, and (3) the causes of illness. The instrument was adapted to Turkish by Kocaman et al. (2007). The “illness identity” subscale consists of 14 items on symptoms (pain, burning in the throat, nausea, difficulty breathing, weight loss, fatigue, joint stiffness, burning eyes, wheezing, headache, stomach complaints, dizziness, difficulty sleeping, and loss of strength). For each of these, the respondent is first asked “whether the symptom was encountered since the beginning of the disease” and then “whether this symptom is related to the disease.” This subscale is organized in such a format that both questions can be replied to as yes/no. The sum of the “yes” replies to the second question was the total score of this subscale.

The “perceptions about the illness” subscale comprises 38 items rated on a five-point Likert-type scale. This subscale has seven subcategories: (1) timeline (acute/chronic), (2) consequences, (3) personal control, (4) treatment control, (5) illness coherence, (6) timeline (cyclical), and (7) emotional representations. The “causes of illness” subscale consists of 18 items rated on a five-point Likert-type scale. This subscale has four subcategories: (1) psychological attributions, (2) risk factors, (3) immunity, and (4) accidents/chances. For qualitative evaluation, the respondent must also write down three factors as the most important causes of his/her illness. The items are rated on a scale ranging from 1 (strongly disagree) to 5 (strongly agree). The total score is calculated by summing all the scores. A lower total score is interpreted negatively in certain dimensions of illness perception [such as timeline (acute-cyclical), personal and treatment control], while it is interpreted positively in other dimensions [consequences, illness coherence, emotional representations, illness identification]. In the last subscale, if symptoms of the disease are listed at the time of diagnosis and are attributed to the disease, each symptom receives a score of 1 point. The “illness identity,” “perceptions about the illness,” and “the causes of illness” subscales had Cronbach’s alpha values of 0.89, 0.69-0.77, and 0.25-0.72, respectively. In this study, the subscales had Cronbach’s alpha values of 0.84, 0.14-0.93, and 0.29-0.65, respectively [16-19].

Adaptation to Chronic Illness Scale

The ACIS was developed by Atik and Karatepe [20]. The instrument consists of 25 items rated on a five-point Likert-type scale (1 = strongly disagree to 5 = strongly agree). The instrument has three subscales: (1) physical adaptation, (2) social adaptation, and (3) psychological adaptation. The total score ranges from 25 to 125, with higher scores indicating better disease adaptation. The scale has a Cronbach’s alpha of 0.88, which was 0.73 in this study [20].

Ethical considerations

The study was approved by the Osmaniye Korkut Ata University Scientific Research and Publication Ethics Committee (30.09.2021-2021/6/11).

Analysis

The data were analyzed using the IBM SPSS Statistics for Windows, Version 15 (Released 2012; IBM Corp., Armonk, New York) at a significance level of 0.05. Continuous data are presented as the mean ± standard deviation (SD) and median (minimum-maximum). The frequency (n) and percentage (%) were calculated for categorical variables. One-way ANOVA and Student’s t-test were used to compare the groups. Tukey’s test was used for post hoc pairwise comparisons to determine the source of difference. Pearson’s correlation coefficients were calculated for two continuous variables.

## Results

Table 1 shows the sociodemographic characteristics of the participants. The participants had a mean age of 54 ± 7.5 years and a mean BMI of 28.3 ± 4 kg/m². More than half of the participants were women (60.9%, n = 207). Most participants were married (87.4%, n = 297). More than half of the participants had a primary school degree (54.4%, n = 185). More than half of the participants were housewives (51.5%, n = 175). More than half of the participants lived with their spouses and children (57.6%, n = 196). Less than half of the participants were smokers (32.1%, n = 109). Most participants did not consume alcohol (93.5%, n = 318). Less than half of the participants exercised thrice a week for 30 minutes (Table 1).

**Table 1:** Personal characteristics of individuals with diabetes (n=340)

Descriptive features		Values
Age (year), mean±SD	54.03±7.05	
BMI, mean±SD	28.30±4.00	
Gender, N (%)		
Female		207 (60.9)
Male		133 (39.1)
Marital status, N (%)		
Single		43 (12.6)
Married		297 (87.4)
Education, N (%)		
Illiterate		28 (8.2)
Primary education		185 (54.4)
High school		93 (27.4)
University		34 (10.0)
Job, N (%)		
Housewife		175 (51.5)
Officer		22 (6.5)
Employee		50 (14.7)
Retired		93 (27.4)
Person living with, N (%)		
Wife		95 (27.9)
Wife and children		196 (57.6)
Siblings, parents		11 (3.2)
Their children		25 (7.4)
Only		13 (3.8)
Smoking, N (%)		
Yes		109 (32.1)
No		231 (67.9)
Alcohol consumption, N (%)		
Yes		22 (6.5)
No		318 (93.5)
Exercise practice, N (%)		
Yes		132 (38.8)
No		208 (61.2)

Table 2 shows the clinical characteristics of the patients. Participants were diagnosed with DM approximately nine years ago (8.6 ± 5.4 years). More than half of the participants had chronic diseases other than DM (56.8%, n = 193). Most participants knew about integrative interventions (84.1%, n = 286). Less than half of the participants learned about integrative interventions from friends (45.9%, n = 156). Less than a quarter of the participants had turned to integrative interventions (23.5%, n = 80), such as cupping therapy (7.6%, n = 26) or cinnamon therapy (7.1%, n = 24) (Table 2).

**Table 2:** Distribution of clinical characteristics of individuals with diabetes mellitus (DM) (n=340)

Descriptive features		Values
Diagnosed with DM (year), Mean±SD	8.62±5.48	
Do you have a concomitant chronic disease? (N(%))		
Yes		193 (56.8)
No		147 (43.2)
Do you know about integrative interventions? (N(%))		
Yes		286 (84.1)
No		54 (15.9)
If yes		
TV-media		41 (12.1)
Family		89 (26.2)
Friend		156 (45.9)
Have you applied to integrative interventions due to your illness? (N(%))		
Yes		80 (23.5)
No		260 (76.5)
If yes		
Cupping		26 (7.6)
Lemon juice		9 (2.6)
Garlic		6 (1.8)
Dandelion		3 (0.9)
Turkish coffee		3 (0.9)
Olive leaf		6 (1.8)
Cinnamon		24 (7.1)
Pomegranate syrup		3 (0.9)
Do you think the integrative interventions you applied were effective? (N(%))		
Yes		75 (22.1)
No		5 (1.5)

Table 3 shows the scale scores. Participants had mean SWBS and ACIS scores of 118.40±11.46 and 84.46±9.18, respectively, and a mean IPQ “illness identity” subscale score of 24.21±5.2. The participants had the highest score on the “timeline (acute/chronic)” subcategory of the “perceptions about the illness” subscale. The participants scored lowest on the “timeline (cyclical)” subcategory of the “perceptions about the illness” subscale. The participants had the highest score on the “psychological attributions” subcategory of “the causes of illness” subscale. The participants scored lowest on the “accident/chance” subcategory of “the causes of illness” subscale (Table 3).

**Table 3:** Scale total scores of individuals with diabetes (n=340) SD: standard deviation; Min: minimum; Max: maximum.

	Mean±SD	Min-Max
SWBS (Spiritual Well-Being Scale)	118.40±11.45	85-145
ACIS (Adaptation to Chronic Illness Scale)	84.46±9.18	58-110
IPQ (Illness Perception Questionnaire)		
Illness identity	3.98±2.58	0-13
Perceptions about the illness		
Timeline (acute/chronic)	24.21±5.2	6-30
Consequences	20.08±3.86	8-29
Personal control	23.86±3.08	16-30
Treatment control	18.73±2.60	10-25
Illness coherence	15.34±4.90	5-25
Timeline (cyclical)	13.38±2.3	4-20
Emotional representations	20.44±4.10	7-30
Causes of illness		
Psychological attributions	20.25±3.91	8-28
Risk factors	18.80±3.71	7-29
Immunity	6.24±2.07	3-12
Accident/chance	3.52±1.30	2-8

Table 4 shows the correlations between the SWBS and ACIS subscale scores. There was a positive correlation between the SWBS and ACIS total score (R= 0.337, p=0.000).

**Table 4:** Correlations between the SWBS and ACIS subscale scores The data have been presented as R and P values. **Correlation is significant at the p<0.01 level (2-tailed). *Correlation is significant at the p<0.05 level (2-tailed).

ACIS subscale	SWBS subscale						SWBS (total)	
	Transcendence		Harmony with nature		Anomie			
	R	P	R	p	R	p	R	p
Physical adaptation	0.312^**^	0.000	0.305^**^	0.000	0.097	0.073	0.311^**^	0.000
Social adaptation	0.266^**^	0.000	0.261^**^	0.000	0.156^**^	0.004	0.292^**^	0.000
Psychological adaptation	0.125^*^	0.021	0.087	0.110	0.111^*^	0.041	0.141^**^	0.009
ACIS (total)	0.321^**^	0.000	0.303^**^	0.000	0.156^**^	0.004	0.337^**^	0.000

Table 5 shows the correlations between the IPQ, ACIS, and SWBS subscale scores. There was a positive correlation between the SWBS total score and the IPQ “timeline (acute/chronic)” subcategory score (R = 0.175, p = 0.001). There was also a positive correlation between the SWBS total score and the IPQ “personal control” and “treatment control” subcategory scores (R = 0.158, p = 0.003; R = 0.173, p = 0.001). This indicates that participants with more personal and treatment control had greater spiritual well-being. There was a negative correlation between the SWBS total score and the IPQ “emotional representations” subcategory score (R = -0.172, p = 0.001). Emotional representations relate to how much a patient thinks he or she would be emotionally affected by the illness. A high score in the “emotional representations” subcategory is interpreted negatively. Therefore, patients with higher levels of spiritual well-being are emotionally positively affected. There was a negative correlation between the ACIS total score and the IPQ “consequences,” “timeline” (acute/chronic, cyclical), and “emotional representations” subcategory scores (R = -0.234, p = 0.000; R = -0.132, p = 0.015; R = -0.308, p = 0.000). The timeline (acute/chronic, cyclical) relates to patients' perception of the disease as acute or chronic and of symptoms as permanent or transient. Consequences concern how the disease affects an individual's quality of life and functioning. Patients who thought that their disease duration would increase and that their symptoms would be permanent had higher mean adaptation scores. As the level of adaptation to chronic diseases increases, patients experience positive effects, as this elevation is linked to reduced emotional impact and less disruption of their daily lives. There was a positive correlation between the ACIS total score and the IPQ “personal control,” “treatment control,” and “illness identification” subcategory scores (R = 0.309, p = 0.000; R = 0.239, p = 0.000; R = 0.340, p = 0.000). These results indicate that DM patients who adapt to their disease have more personal and treatment control and know less about it and its symptoms. There was a negative correlation between the ACIS total score and the IPQ “immunity” and “accident/chance” subcategory scores (R = -0.186, p = 0.001; R = -0.165, p = 0.002). These results indicate that DM patients who adapt to their disease tend to view conditions affecting immunity (e.g., viruses) and accidents/bad luck as causes of illness less often.

**Table 5:** Correlations between IPQ, ACIS, and SWBS subscale scores The data have been presented as R and P values. **Correlation is significant at the p<0.01 level. *Correlation is significant at the p<0.05 level (2-tailed). IPQ: Illness Perception Questionnaire, ACIS: Adaptation to Chronic Illness Scale, SWBS: piritual Well-Being Scale.

IPQ subscale scores	SWBS subscale scores						SWBS total		ACIS subscale scores						ACIS total	
	Transcendence		Harmony with nature		Anomie				Physical adaptation		Social adaptation		Psychological adaptation			
	R	p	R	p	R	p	R	p	R	p	R	p	R	p	R	p
Illness identity	-0.024	0.653	0.038	0.491	-0.010	0.853	-.007	0.904	-0.107^*^	0.049	-0.030	0.580	0.019	0.730	-0.063	0.248
Perceptions about the illness																
Timeline (acute/chronic)	0.183^**^	0.001	0.173^*^	0.001	0.044	0.419	0.175^**^	0.001	0.092	0.090	-0.036	0.514	-0.335^**^	0.000	-0.074	0.174
Consequences	0.013	0.809	-0.019	0.724	0.013	0.811	0.007	0.903	0.009	0.871	-0.311^**^	0.000	-0.311^**^	0.000	-0.234^**^	0.000
Personal control	0.158^**^	0.003	0.102	0.061	0.095	0.082	0.158^**^	0.003	0.305^**^	0.000	0.218^**^	0.000	0.167^**^	0.002	0.309^**^	0.000
Treatment control	0.088	0.106	0.151^**^	0.005	0.205^**^	0.000	0.173^**^	0.001	0.234^**^	0.000	0.063	0.247	0.287	0.000	0.239^**^	0.000
Illness coherence	0.017	0.756	0.060	0.271	0.110^*^	0.043	0.069	0.205	0.229^**^	0.000	0.245^**^	0.000	0.351^**^	0.000	0.340^**^	0.000
Timeline (cyclical)	0.024	0.658	0.087	0.110	-0.005	0.924	0.039	0.479	-0.016	0.766	-0.105	0.053	-0.241^**^	0.000	-0.132^*^	0.015
Emotional representations	-0.133^*^	0.014	-0.101	0.063	-0.171^**^	0.002	-0.172^**^	0.001	-0.080	0.143	-0.300^**^	0.000	-0.416^**^	0.000	-0.308^**^	0.000
Causes of illness																
Psychological attributions	0.108^*^	0.046	0.128^*^	0.018	-0.227^**^	0.000	0.018	0.745	0.103	0.059	-0.051	0.349	-0.133^*^	0.014	-0.012	0.826
Risk factors	-0.050	0.361	0.016	0.764	-0.018	0.735	-0.031	0.571	-0.028	0.612	-0.073	0.180	-0.133^*^	0.014	-0.089	0.101
Immunity	-0.049	0.369	0.017	0.761	-0.038	0.479	-0.038	0.488	-0.198^**^	0.000	-0.105	0.053	-0.113^*^	0.038	-0.186^**^	0.001
Accident/chance	-0.144^**^	0.008	-0.149^**^	0.006	-0.098	0.070	-0.165^**^	0.002	-0.148^**^	0.006	-0.175^**^	0.001	-0.030	0.586	-0.165^**^	0.002

## Discussion

This study investigated the relationships between spiritual well-being and illness perception and disease adaptation. The results showed that participants had good physical, social, and psychological adaptation to their chronic diseases. Ustaalıoglu and Tan reported that adults with DM had positive attitudes toward care and treatment [21]. İnel Manav et al. conducted a study investigating the levels of unconditional self-acceptance among adults with DM and their degree of adaptation to the disease [2]. The findings indicated that participants effectively adapted to the disease. Kaymaz and Akdemir documented that patients with DM had moderate psychosocial adaptation to the disease [22]. On the other hand, Çelik et al. reported that almost all patients with DM had poor psychosocial adaptation to their disease [23]. Adapting to diabetes, which necessitates the development of numerous new attitudes and perceptions, is a complex and multifaceted challenge for patients [2,24]. Patients with DM who receive support from healthcare professionals and caregivers adapt to the disease more easily.

Our participants had above-average spiritual well-being. While a considerable amount of related research has been dedicated to exploring the spiritual well-being of individuals with chronic diseases, only a few researchers have specifically examined the spiritual well-being levels of patients with DM. Javanmardifard et al. also reported that individuals with DM had moderate spiritual well-being [25]. Individuals with DM with high levels of spiritual well-being tend to experience a greater sense of empowerment, enabling them to manage factors that could challenge their overall well-being effectively. This empowerment also supports their ability to engage in everyday activities successfully. In contrast to other medical conditions that may involve medication, diabetes management entails dealing with a more intricate interplay of physiological, psychological, and social factors. This complexity renders the management process exceptionally challenging. Additionally, successful diabetes management demands physical effort and a significant emphasis on spiritual and psychological aspects. This holistic approach is crucial for effectively implementing lifestyle modifications, including weight loss, dietary adjustments, and regular exercise. These lifestyle changes are fundamental to diabetes treatment and rely heavily on the patient's commitment and active participation. 

Spiritual well-being plays a pivotal role in fostering a positive outlook toward the daunting life experiences that arise from diabetes. It contributes to an improved life experience by instilling motivation and vitality. This is attributed to enhancing psychological functioning and adaptability, bolstering various dimensions of health, and elevating overall quality of life. These effects have been observed through various studies [25,26]. Spiritual well-being enhances an individual's capacity for tolerance and acceptance of unalterable conditions, especially when medical interventions fail to achieve the desired outcomes for patients with DM [27-29]. We also observed a positive relationship between spiritual well-being and disease adaptation. Yılmaz and Kara focused on patients with chronic diseases, including DM, and reported that participants with higher spiritual well-being adapted better to their diseases [30]. Gupta and Anandarajah conducted a qualitative study examining the impact of spirituality on disease management [31]. The study revealed that participants held more positive perceptions about their illness, as they believed that divine intervention by a higher power, such as God, played a role in assisting them [31].

Our participants perceived their illness as chronic. Our results also indicated a positive relationship between spiritual well-being and disease perception. In addition, participants with higher spiritual well-being had fewer negative effects related to their illness and were better at controlling their disease and benefitting from its treatment. Holt-Lunstad et al. also reported that higher spiritual well-being was associated with lower triglyceride levels and fasting glucose [32]. Zareipour et al. documented that older diabetic individuals with greater mental health had lower blood glucose levels [33]. Javanmardifard et al. reported that DM patients with greater spirituality had lower HBA1c levels [25]. The fact that our participants had moderate spiritual well-being suggested that spirituality serves as a source of empowerment and support for individuals with diabetes, aiding them in embracing the enduring nature of the condition. It assists in managing the disease, fostering acceptance, and cultivating a constructive mindset toward the illness.

Lifestyle changes and long-term treatment and care are needed for patients with diabetes. The symptoms of diabetes render patients susceptible to emotional stressors, a circumstance linked to reduced treatment adaptation and poorer health outcomes [34,35]. Our findings indicated that participants who demonstrated greater consistency in their attitudes toward the disease reported lower levels of negative affect, and held perceptions of reduced negative consequences associated with the condition displayed an inclination toward improved adaptation to diabetes management. In addition, participants who possessed a comprehensive understanding of their disease effectively managed their symptoms and believed that treatment would yield benefits exhibited higher levels of adaptation. Research shows that individuals with heightened perceptions of their illness tend to hold more favorable viewpoints about medical treatment and demonstrate elevated adaptation to illness-related treatments [36,37]. Bilondi et al. noted that patients diagnosed with type 2 diabetes who possessed a clearer perception of their disease exhibited a heightened sense of responsibility for their well-being and demonstrated enhanced adaptation to their treatment protocols [38]. Ross et al. found a correlation between patients’ illness perceptions and treatment and management adaptation [39]. We believe that spiritual well-being equips DM patients with emotional resilience, empowering them to cultivate optimistic perspectives regarding their disease.

## Conclusions

DM patients with elevated spiritual well-being exhibit an improved ability to adapt to their disease. Moreover, DM patients with more positive perceptions about their disease tend to demonstrate enhanced abilities in adapting to their condition, often coinciding with higher levels of spiritual well-being. In line with these results, we can state that it is crucial to educate individuals with diabetes about their individual risk factors, raise awareness about available spiritual resources, and underscore the significance of viewing spiritual well-being as a coping mechanism to aid them in adapting to their condition.
